# An Integrated Assessment Approach to Address Artisanal and Small-Scale Gold Mining in Ghana

**DOI:** 10.3390/ijerph120911683

**Published:** 2015-09-17

**Authors:** Niladri Basu, Elisha P. Renne, Rachel N. Long

**Affiliations:** 1 Faculty of Agricultural and Environmental Sciences, McGill University, 21, 111 Lakeshore Rd., Ste. Anne de Bellevue, Quebec H9X 3V9, Canada; 2 Department of Environmental Health Sciences, School of Public Health, University of Michigan, 1415 Washington Heights, Ann Arbor, MI 48109, USA; E-Mail: rachlong@umich.edu; 3 Department of Anthropology, University of Michigan, 101 West Hall, Ann Arbor, MI 48109, USA; E-Mail: erenne@umich.edu; 4 Department of Afro-American and African Studies, University of Michigan, 4700 Haven Hall, Ann Arbor, MI 48109, USA

**Keywords:** Minamata Convention, implementation, policy, mercury, public health, anthropology, history, social sciences, economics, gender, small-scale mining, artisanal mining, gold mining

## Abstract

Artisanal and small-scale gold mining (ASGM) is growing in many regions of the world including Ghana. The problems in these communities are complex and multi-faceted. To help increase understanding of such problems, and to enable consensus-building and effective translation of scientific findings to stakeholders, help inform policies, and ultimately improve decision making, we utilized an Integrated Assessment approach to study artisanal and small-scale gold mining activities in Ghana. Though Integrated Assessments have been used in the fields of environmental science and sustainable development, their use in addressing specific matter in public health, and in particular, environmental and occupational health is quite limited despite their many benefits. The aim of the current paper was to describe specific activities undertaken and how they were organized, and the outputs and outcomes of our activity. In brief, three disciplinary workgroups (Natural Sciences, Human Health, Social Sciences and Economics) were formed, with 26 researchers from a range of Ghanaian institutions plus international experts. The workgroups conducted activities in order to address the following question: What are the causes, consequences and correctives of small-scale gold mining in Ghana? More specifically: What alternatives are available in resource-limited settings in Ghana that allow for gold-mining to occur in a manner that maintains ecological health and human health without hindering near- and long-term economic prosperity? Several response options were identified and evaluated, and are currently being disseminated to various stakeholders within Ghana and internationally.

“Be contented and turn your eyes to what I shall present you concerning the Gold itself…..This Illustrious Metal is generally found in three sorts of places: First, the best in found in or betwixt particular Hills; and apprehending where the Gold, [they] dig Pits; and separate it from the Earth which comes out with it. The second place is in, at, and about some Rivers and Water-falls; whose violence washeth down great quantities of Earth, with carry the Gold with it. The third is on the Sea shore; where (as at Elmina and Axim) there are little Branches or Rivulets into which the Gold is driven from Mountainous Places, as well as Rivers…. these places are visited by hundreds of women [who are] furnished with large and small Troughs or Tray[s], which they first fill full of Earth and Sand, which they wash with repeated fresh Water;…if there is any Gold…it is thrown into the small Tray…”

From Willem Bosman, 1705. *A New and Accurate Description of the Coast of Guinea…*, translated from the original Dutch. London: J. Knapton, 1705, pp. 80–81 [1].

## 1. Artisanal and Small-Scale Gold Mining (ASGM) in Ghana

Informal gold mining, referred to as artisanal and small-scale gold mining (ASGM), has proliferated worldwide due to contemporary and powerful economic forces and widespread poverty across low- and middle-income countries. It is estimated that upwards of 15 million people worldwide may directly be involved in ASGM, and another 100 million people may be reliant upon the sector [2,3]. Gold from small-scale mines may represent 20%–30% of the world’s output [4]. 

West Africa, particularly Ghana, is one of the world’s most important gold mining regions. Gold has been mined in Ghana for over 1000 years [5] and, as Bosman has discussed [1], gold mining was well-developed in the Gold Coast by the 16th century. Its production has increased 700% since 1980 and in 2012 gold accounted for 43% of the country’s national exports [6,7]. Ghana is second only to South Africa in gold production from Africa [8]. The ASGM sector is estimated to have increased from 6% of Ghana’s gold production in 2000 to 23% in 2010 [9]. The sector may employ an estimated 1.1 million people (with estimates suggesting that most work illegally) and represent nearly two-thirds of the country’s total mining labor force [10,11] 

The ASGM sector is faced with numerous challenges. Many mining community residents are impoverished and live in rural settings that lack basic resources such as health care services and clean potable water [12]. Mining activities can destroy landscapes, grossly contaminate water resources, and stress water tables [13]. Mercury contamination of water, soil, and food is well documented in ASGM communities, and newer studies have documented that exposures to other toxic elements such as arsenic and lead may be widespread [14]. Many residents have expressed deep concern about access to clean water, but are willing to accept compromised water quality in exchange for income and sustenance [15]. In Ghana, while unlicensed small-scale gold mining is illegal, the government is unable to monitor the activities of *galamsey* miners [16]. *Galamsey* is the term commonly used for illegal small-scale miners [17]. While some *galamsey* are working in areas where they have long resided and practiced traditional gold mining as their legitimate right [18], since they are not registered and do not pay taxes, government officials cannot provide resources to these communities. Small-scale miners’ fear of prosecution exacerbates the situation, fueling this illicit market economy. Furthermore, women, who have long been engaged in small-scale gold mining, face different problems from their male counterparts, particularly concerning prevailing gender roles and reproductive health [19].

The problems in ASGM communities are multi-faceted and complex, and so improvements to the sector can only be realized by considering different approaches and disciplinary perspectives. Here we outline the use of an Integrated Assessment approach to increase understanding of policy and response options concerning ASGM in Ghana. As elaborated upon in the following sections, Integrated Assessments have been used in the realms of environmental science and sustainable development [20], but their use to address specific matters in public health, and in particular, environmental and occupational health, is quite limited. This is despite the numerous benefits they offer as discussed below. This paper also serves as an introduction to a special issue of the International Journal of Environmental Research and Public Health (http://www.mdpi.com/journal/ijerph/special_issues/asgm) entitled “Integrated Assessment of Artisanal and Small-Scale Gold Mining (ASGM) in Ghana”. 

## 2. A Primer on Integrated Assessments

Integrated Assessments have been defined as:

“a way of bringing together knowledge of ecosystems, people, and policy in order to find solutions for particularly challenging problems and to develop tools and information that policy makers can use.” [21]; 

“a collective, deliberative process by which experts review, analyze, and synthesize scientific knowledge in response to user’s information needs relevant to key questions, uncertainties or decisions” [22]; “policy-motivated research to develop an understanding of the issue, not based on disciplinary boundaries but based on boundaries defined by the problem” [23].

A major activity of an Integrated Assessment is to identify and assess best available information using diverse and multi-disciplinary approaches, so as to enable consensus-building and effective translation of scientific findings to stakeholders, help inform policies, and ultimately improve decision making [20]. An Integrated Assessment aims to bring together key aspects and goals of other assessment methods including Process Assessments (which describe a process and evaluate status, trends, and underlying causes), Impact Assessments (which identify and assess the possible consequences of an environmental problem towards human health, ecosystems, and socioeconomic systems), and Response Assessments (which identify and evaluate the possible responses, including policies, to an environmental problem). 

**Figure 1:**
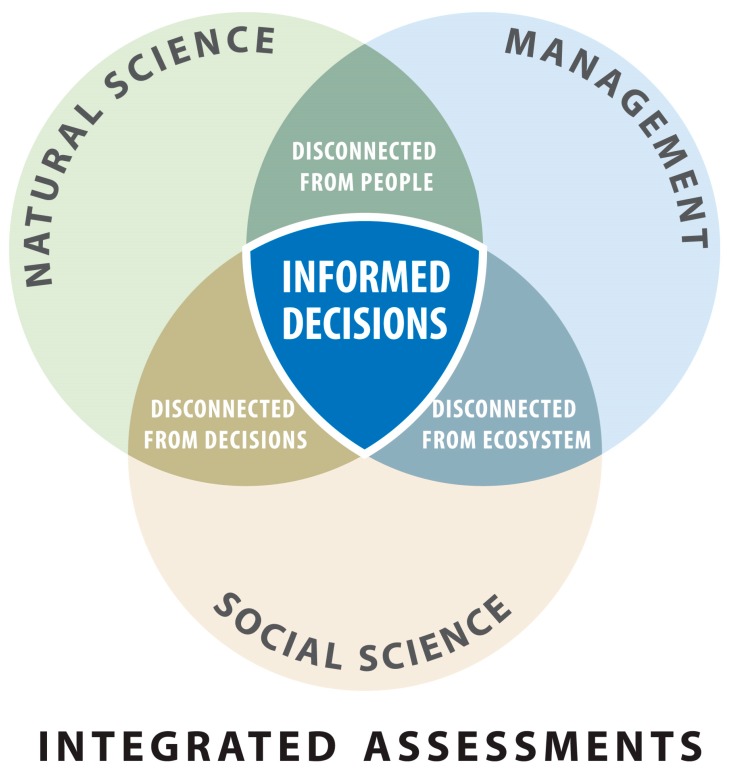
An Integrated Assessment brings together groups normally separated along disciplinary or sectoral lines to facilitate multi-disciplinary dialogue and work towards building consensus. Figure is reproduced with permission from the Michigan Sea Grant.

There are several benefits to tackling complex problems, particularly those lacking a clear cause or solution, using an Integrated Assessment framework. According to a document published by NOAA [24], Integrated Assessments can help to produce outcomes or decisions that have higher likelihood of success. The process includes a number of steps in which response options and policy actions, as well as their forecasted impacts, are evaluated iteratively. Often they are conducted in the context of a specific management of policy issue, and thus they have a clear end user. By bringing together groups normally separated along disciplinary (e.g., natural sciences and social sciences) or sectoral (e.g., industry and government) lines, they can facilitate multi-disciplinary dialogue and work towards building consensus. For example, as depicted in Figure 1, social scientists and natural sciences are often disconnected from policies, yet informed decisions require the involvement of those with a policy background. By engaging with diverse stakeholders, and especially the public, the Integrated Assessment process helps enable broad support due to its consultative nature, facilitation of local knowledge into decision-making practices, and advancement of public understanding of the issue. The process also creates new relationships amongst participants who may not normally interact. 

## 3. Integrated Assessment to Address ASGM in Ghana

**Figure 2:**
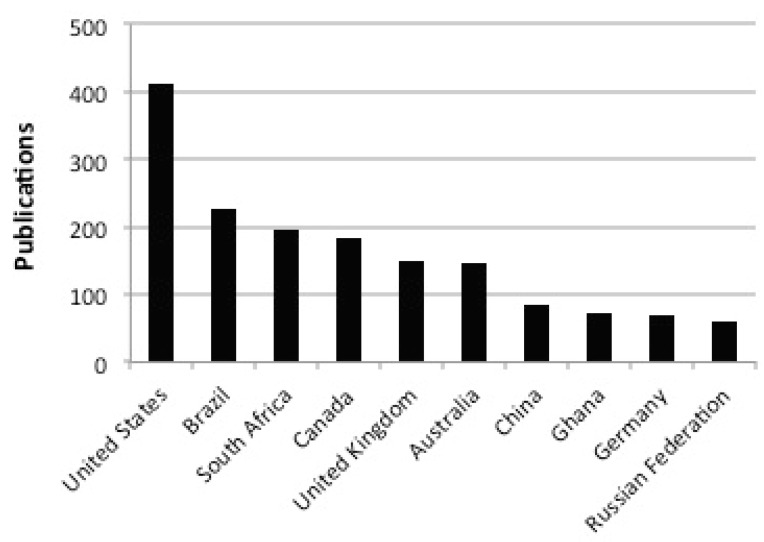
A simple bibliometric analysis using Scopus reveals that Ghanaian researchers are amongst the most prolific worldwide. On 19 March (2015), the term “gold mining” was searched resulting in 2531 papers. Research hailing from Ghana was in the top 10 of all countries.

Conducting the activity in Ghana made sense for many reasons. Foremost is that the Co-Principal Investigators have been working in Ghana since 2006, and ASGM was amongst the various topics under investigation [15,19,25,26]. Over time relationships had been formed with stakeholders including community residents, mine concession owners, government officials, and researchers from across academia, NGOs, and the government. Ghana also represented an excellent platform for this work owing to a relatively large group of in-country researchers who have published widely on ASGM in their own country. A simple bibliometric analysis revealed that Ghanaian researchers are amongst the most prolific worldwide on the topic of ASGM. A search in Scopus on 19 March 2015 for the term “gold mining” resulted in 2531 papers, and research hailing from Ghana was in the top 10 of all countries (Figure 2). The Integrated Assessment process afforded an unprecedented opportunity to bring together several of these active researchers, many of whom had not previously met. 

## 4. Approach and Activities

From its conception the project was carefully designed to bring together interested Ghanaian ASGM researchers. To ensure that critical perspectives were captured and to align with the Integrated Assessment framework, three disciplinary workgroups were formed: Natural Sciences, Human Health, and Social Sciences and Economics. For each workgroup, co-leaders were identified and immediately engaged. The co-leaders consisted of at least one researcher from Ghana paired with at least one researcher from the University of Michigan. All of the co-leaders had prior collaborative history and thus had developed collegial relationships. 

The co-leaders had two initial tasks: first, they developed and refined the policy relevant question that would guide the Integrated Assessment. Following several discussions over email and meetings in-person, the group decided to organize the Integrated Assessment to tackle the question: *What are the causes, consequences and correctives of small-scale gold mining in Ghana?* More specifically: *What alternatives are available in resource-limited settings in Ghana that allow for gold-mining to occur in a manner that maintains ecological health and human health without hindering near- and long-term economic prosperity?*

The second initial task of the co-leaders was to identify workgroup members. It was necessary to ensure that each group consisted of at least five members who hailed from different institutions and/or sectors government, academia, NGO), and to also ensure that they were mainly Ghanaian researchers. As mentioned earlier, a great benefit of pursuing research activities in Ghana is the great in-country expertise concerning ASGM (Figure 2). In total, 26 workgroup members were recruited, hailing from a range of institutions including: the Ministry of Lands and Resources; the Ghana Health Service; the Water Research Institute (at the Council for Scientific and Industrial Research); the Centre for Energy, Environment, and Sustainable Development; Kwame Nkrumah University of Science and Technology; University of Ghana; University for Development Studies; University of Cape Coast; Institute for Development Studies; Kumasi Polytechnic; University of Michigan; and McGill University. In the initial stages of the work we purposefully limited membership to active scientific researchers and thus did not include other important stakeholders such as miners and policy makers. This was to ensure that we focused on the scientific evidence, but also had confidence that other perspectives were infiltrating into the initial phases given that all the researchers involved work intimately with other stakeholder groups. Further, the latter stages of the project (such as Meeting #4) involved dissemination and exchange with relevant stakeholders.

The overall project was designed to be 40 months in duration (May 2012 through August 2015) with several milestones pre-identified. The major activities include a series of workshop meetings held in Accra (Table 1) coupled with regular exchanges via email, both carefully orchestrated to shepherd the workgroups through the key Integrated Assessment steps (Table 2). To facilitate open dialogue, workshops included a range of activities including, for example, guided and unstructured brainstorming sessions, individual researcher updates, and iterative polls using paper and electronic surveys. During these meetings researchers mainly from Ghana delivered 37 presentations. The core activities concerned the identification and scrutiny of datasets so as to document the status, trends, causes, and consequences of ASGM in Ghana (Step 2 in Table 2). This was achieved via in person meetings (Meetings #2 and #3) and online exchanges for a period of approximately 1.5 years. Related to this, the process has facilitated the researchers further developing their own datasets and findings into publications that are being published in this special issue of the International Journal of Environmental Research and Public Health (http://www.mdpi.com/journal/ijerph/special_issues/asgm). The second main activity was the identification and evaluation of response options (Steps 3 and 4 in Table 2). As elaborated upon below, this activity entailed several exchanges within Workgroups to develop a list of 12 response options that were justified based on the reviewed evidence. During Meeting #3, the Workgroups presented their options to the entire delegation who then provided feedback and evaluative scores. Meeting #3 ended with a list of the ranked list of response options, of which the top six were presented to decision makers and stakeholders (38 individual representing 28 Institutions) at Meeting #4.

**Table 1:** Outline of meeting activities as part of our integrated assessment.

Meeting #	Dates	Participants (# in Brackets)	Key Activities
1	6–7 August 2012	Organizer (1), Co-Leaders (9)	policy relevant question defined workgroup members and stakeholders identified
2	11–12 April 2013	Organizer (1), Co-Leaders (9), Workgroup Members (11)	22 research presentations delivered by individuals workgroup breakout sessions
3	28–30 April 2014	Organizer (1), Co-Leaders (9), Workgroup Members (13), External Advisors (4), Facilitators (2), Observer from Funding Agency (1)	3 summary presentations on each workgroup’s findings and recommended response options Delphi poll used by researchers to refine and evaluate response options
4	21–22 April 2015	Organizer (1), Co-Leaders (3), Facilitators (2), External Advisors & International Stakeholders (5), Decision Makers (25)	summary presentations on findings and recommended response options Delphi poll used by stakeholders to refine and evaluate response options focused discussion on implementation of the Minamata Convention

**Table 2:** Key steps of the integrated assessment.

Brief Overview of Integrated Assessment Steps	
1	Define policy-relevant question
2	Document status/trends; causes/consequences
3	Identify desired outcomes/policy options
4	Evaluate policy options
5	Provide technical guidance for implementation
6	Assess uncertainty
7	Evaluation
8	Submit findings for peer review and public comment

The organization of the meetings was carefully considered so as to promote open discussion and active participation. Meals and break periods enabled free-flowing unstructured discussions and provided networking opportunities, and this dedicated time was critical to establishing and growing relationships. Event scheduling occurred several months in advance to ensure that all participants could be engaged and that dates did not conflict with other events. All participants were afforded an opportunity to help set the agenda (*i.e*., sent draft agendas prior to the meeting to comment upon), and were provided all materials in advance of the meeting. Time was effectively moderated so as to respect participants’ time. Workgroup members, especially those from the global North, were cognizant of the need to listen and enable open discussion, rather than dominate conversation. The meeting was designed to provide a neutral and open forum. This was continuously discussed by the organizers and queried in the yearly evaluations. For the final two meetings neutral facilitators (from Ghana’s Science and Technology Policy Research Institute, STEPRI) were brought into the process.

Engagement was also promoted by constantly reminding the workgroup members of the overall Integrated Assessment process, and how their activities both within their workgroups but also as individual researchers (*i.e*., publication of their individual research papers; involvement in future grant applications) would feed into the larger process. Careful notes were taken during each meeting and promptly shared with all participants within two weeks of each meeting. Between meetings there was constant communication amongst workgroup members and the larger team. A website (asgmresearch.weebly.com) served as a clearinghouse of information, and advertised our work internally and to outside stakeholders. 

## 5. Key Findings from the Integrated Assessment

The results of the Integrated Assessment are being published in this special issue of the International Journal of Environmental Research and Public Health (http://www.mdpi.com/journal/ijerph/special_issues/asgm). In addition to this introductory piece, each of the three workgroups has published a detailed assessment report [12,13,14], and there is a paper outlining the application of the Delphi method [27,28] to the identification of response options, and 12 individual research papers written by team members on a variety of ASGM matters. Here we provide very brief summaries of the assessment reports written by the three working groups. 

The Human Health report [14] provides evidence from across multiple Ghanaian ASGM sites that documents relatively high exposures to mercury [29,30] and other heavy metals, occupational injuries [31,32,33] and noise exposure [34]. The work also reviews limited data on psychosocial health, nutrition, cardiovascular and respiratory health, sexual health, and water and sanitation. The top three recommended response options from this workgroup were (note that the final three options listed below were tied):

- That registration of small-scale miners be increased by improving the process by, for example, reducing or eliminating fees and localizing registration.
- That universities, the EPA, and the Minerals Commission explore and implement high-yield mercury free alternatives.
- That ministries, local governments, and District Assemblies promote diversification of economic opportunities.
- That the government provide water, electricity, telecommunications, and sanitation in partnership with enterprises to ASGM communities and other affected communities.
- That a national framework for policy and planning implementation be established (i.e., taskforces, workgroups) that considers stakeholder input.

The Natural Sciences Report [13] documents evidence of mercury contamination in a range of ecological media, including soil, foodstuffs, sediment, and water [29,35,36,37]. Other water quality parameters near ASGM sites show impairment, with some samples exceeding guidelines for acidity, turbidity, and nitrates. Deforestation and land degradation often accompany ASGM activity, potentially decreasing biodiversity, farmland, and soil fertility. While not well-documented, effects from and on climate change as well as contamination from mining waste may add additional stress on environmental quality and ecosystem services. The top three recommended response options from this workgroup were:

- That the royalties from the proceeds of mining be placed into a central account and directed towards improving health and environment of ASGM communities.
- That a national framework for policy and planning implementation be established (i.e., taskforces, workgroups) that considers stakeholder input.
- That there be public and private support for education with ASG miners on ecological and human health risks, mercury and metals, mercury reduction strategies, and business practices.

The Socioeconomics Report [12] discusses sustainable ASGM development in terms of the legalization and formalization of small-scale gold miners’ activities. The costs of obtaining mineral rights and permits, as well as the related costs of travel and work time lost, contribute to miners’ calculations concerning the perceived value of registering as a legal gold miner. Yet registration is fundamental to miners’ investing in sustainable and environmentally-sound ASGM practices. However, the enforcement of regulations has to be consistent and fair, with rules that are simple, unequivocal and broadly accepted. The authors suggest that to improve stability and sustainability of the ASGM industry, the government should enhance capacity to locally certify and track ASGM activities before enforcing sanctions against non-compliant miners [11]. Such certification and tracking is likely to enhance the long-term stability of ASGM economic benefits and human development potential, while minimizing the adverse health and environmental impacts that need to be addressed. The top three recommended response options from this workgroup were:

- That registration of small-scale miners be increased by improving the process by, for example, reducing or eliminating fees and localizing registration.
- That a national framework for policy and planning implementation be established (i.e., taskforces, workgroups) that considers stakeholder input.
- That ministries, local governments, and District Assemblies promote diversification of economic opportunities.

## 6. Identification of Response Options and the Policy Landscape

One of the major expectations of the Integrated Assessment was that teams would identify response options, or actions to address the social, economic, health and environmental issues associated with ASGM, that could be acted upon by stakeholders. Groups were provided minimal guidance on the development of response options, and were asked only to ensure that there was some evidence supporting the response options developed. 

A detailed description of the entire development and evaluation of the response options can be found in a companion paper [28]. In brief, the response options were developed by workgroups in advance of Meeting #3, and described in the final section of their respective discipline-specific reports. Before the meeting, team leaders reviewed these response options and condensed them into 18 options that were shared with all participants via an online poll (SurveyMonkey.com). At the meeting, workgroups gave brief presentations on their findings, and then elaborated on the response options developed in the reports. Response options were then discussed among the entire group and evaluated via two rounds of polling based on the Delphi technique [28]. 

The current Integrated Assessment is timely for many reasons. Internationally, in 2013, the member nations of the United Nations Environment Program (UNEP) agreed on the Minamata Convention on Mercury. The Convention has both a stand-alone article (Article 7) and a dedicated Annex (Annex C) concerning the ASGM sector. Notably, countries with significant ASGM sectors, such as Ghana, are required to develop a public health strategy for ASGM communities, and take specific measures to protect vulnerable populations such as children and women of childbearing age. The Convention also encourages Parties to cooperate in education, outreach and capacity-building initiatives specific to ASGM (7.4B), as well as engage in general strengthening of public health measures to address mercury pollution (Article 16), especially protection of vulnerable groups (women and children; 16.1A) with specific mention of strengthening of institutional and health professional capacities (16.1D). At the national level, ASGM is built into Ghana’s Minerals and Mining Act, 2006 (Act 703). While many miners are formally registered, it is estimated that about 80% of ASGM miners are not registered and thus conducting the activity illegally (personal communication with Ghana Minerals Commission, 6 August 2014). To help legitimize the sector, in 2013 the President of Ghana launched an Inter-Ministerial Task Force on Small-Scale Mining. In addition, the National Development Planning Commission, which reports to President of Ghana, has recently identified ASGM as a sector of major economic, social and national security concern that requires swift policy action. Efforts are underway to share results of this Integrated Assessment with the aforementioned programs, and other stakeholders.

## 7. Evaluation

A critical feature of the Integrated Assessment was a series of evaluations. The funders of this Integrated Assessment have published a standalone evaluation guideline [37] within which they cite the work of Conley and Moote [38] that state that evaluations “can help: (1) determine when the idealized narrative used to justify collaborative [efforts] holds true; (2) address criticism of these efforts; and (3) assess and refine efforts to institutionalize a movement that has developed largely at the grassroots level.” At the outset of this Integrated Assessment a framework of activities was established with expected outputs and outcomes defined. These are discussed below from an evaluation perspective.

*Technical Adequacy.* To ensure that the work is scientifically sound, it is to be judged via the publication of peer-reviewed papers, generation of factsheets accessible to a range of stakeholders, and the involvement of international experts as external advisors. As mentioned previously a major output of this Integrated Assessment will be the publication of ~18 peer-reviewed papers in this special issue of the International Journal of Environmental Research and Public Health (http://www.mdpi.com/journal/ijerph/special_issues/asgm). Acceptance of these papers by the scientific community will provide strong support of the work’s technical adequacy. We are in the process of finalizing a series of fact sheets as well as data resources that, for example, outline the relevant mining laws in Ghana, provide a table of mercury content in nearly 10,000 specimens (*i.e*., fish, tailings, sediment, plants, soil, water, hair, urine) from across the country, and summarize workgroup findings. These will be made available as hardcopies and also posted on our project website.

*Value and Effectiveness.* The Integrated Assessment is very timely and relevant to ongoing decision making at the international and national levels. As mentioned earlier, Ghana has signed onto the global UNEP Minamata Convention on Mercury that has special provisions for the ASGM sector. At the national level ASGM is built into various Acts and is a focus of an Inter-Ministerial Task Force on Small-Scale Mining and a topic of concern for the National Development Planning Commission. 

Meeting #4 was co-organized by the Principal Investigators along with Ghana’s Science and Technology Policy Research Institute (STEPRI) to disseminate findings to officials (including Ministers, Deputy Ministers, Unit Heads) representing 28 organizations including groups such as the Ministry of Environment, Science, Technology and Innovation (MESTI), the Ministry of Health, the Ministry of Lands and Natural Resources, the National Development Planning Commission (NDPC), the Minerals Commission, the Ministries of Local Government and Rural Development, the Water Research Institute, the University of Ghana School of Public Health, the Ghana Health Service, the University of Mines and Technology, the Environmental Protection Agency, the Ghana Mine Workers’ Union, the Ghana National Association of Small Scale Miners, and the Ghana National Association of Small Scale Businesses. The participants involved in Meeting #4 provided structured and open-ended feedback on the top six response options presented to them, and this is detailed in the companion paper [28].

In addition, the work is warranted given the recent boom in gold prices, global economic crises, expansion of resource development across Africa, and a dearth of information concerning multiple stressors in mining communities. The work also builds off, and thus mutually reinforces, several research grants and research training grants in Ghana (and across West Africa) held by the Co-Principal Investigators and several workgroup members.

The effectiveness of an Integrated Assessment can be determined by its influence on policy. While it is too early to determine this, there are a number of planned activities in coming months that involve key decision makers and managers, both nationally and internationally. Further, the policy landscape concerning ASGM is currently under scrutiny worldwide, and the countrywide assessment conducted here is expected to inform policies in Ghana and potentially elsewhere.

*Legitimacy.* To ensure fairness and ensure an assessment that is balanced and objective, a wide range of stakeholders has been engaged from across disciplines and sectors, including external international advisors. As discussed earlier, meetings were carefully designed so as to enable open dialogue and encourage diverse perspectives. Engagement was tracked via responsiveness to emails and online polls, participation in meetings (both in-person and virtual). 

**Figure 3:**
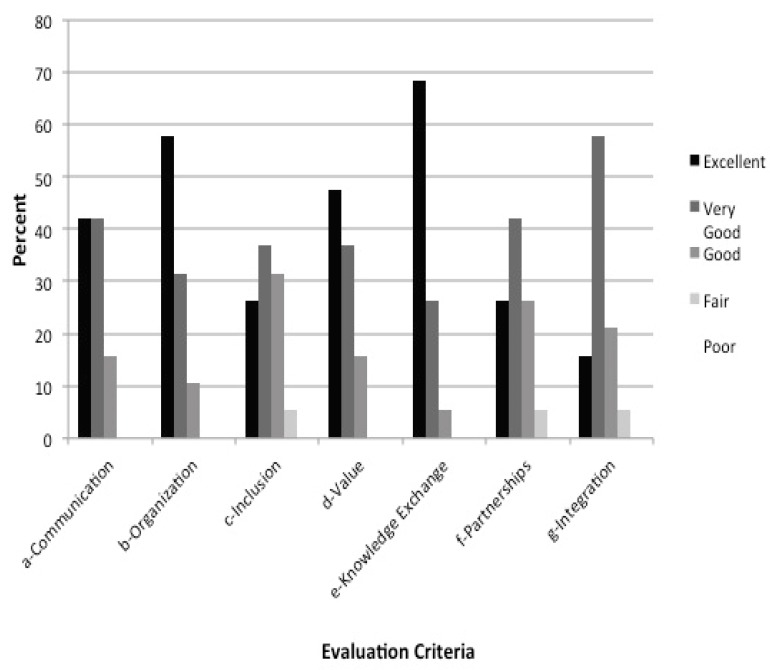
Participant evaluation scores from the third meeting. Scores are representative of other meetings. The evaluation criteria presented here include: A—Clear communication of project goals, activities, and timeline; B—Organization of planning process; C—Inclusion of appropriate stakeholders; D—Value in addressing the problem and research questions; E—Exchange of knowledge; F—Formation of partnerships; G—Integration among diverse stakeholders/experts.

A major scheme by which legitimacy was ascertained was via a series of anonymous surveys of meeting participants. These surveys were administered on paper at the conclusion of each meeting and collected and analyzed by the project manager. The surveys contained both quantitative and open-ended, qualitative questions that addressed both the most recent meeting and the entire Integrated Assessment process. Quantitative questions about the meeting asked about the logistics of the meeting and the meeting’s effectiveness in achieving its stated goals. Quantitative questions about the Integrated Assessment process asked about communication, organization, inclusion of appropriate stakeholders, and the value of the process in addressing the problem, the exchange of knowledge, the formation of partnerships, and integration among diverse stakeholders and experts. Qualitative questions asked for open-ended responses about both positive and negative aspects of the meetings and of the Integrated Assessment process. Figure 3 shows the results of the latest quantitative survey on the Integrated Assessment process, and Table 3 provides some representative responses (pro and con) to qualitative questions. 

**Table 3:** Representative comments of the integrated assessment from the evaluations.

Positive Aspects of the Integrated Assessment Process and/or Meetings
Most impressive were the far reach and depth of discussion of this multifaceted, difficult problem among so many disciplinary perspectives.
The poll strategy was very effective in getting the thoughts of individuals synthesized despite the short period available.
People openly discussed ideas and respected each other’s opinions. Was a sense that people were open to new ways of looking at things.
The [Delphi] poll strategy was very effective in getting the thoughts of individuals synthesized despite the short period available.
Negative aspects of the Integrated Assessment process and/or meetings:
In my opinion, the IA process can be improved by engaging the local communities.
There is the need to identify all relevant stakeholders who are in a position to support the initiative and get the results to influence policy.
Inclusion of policy makers during the process could improve ownership and hence facilitate policy dialogue and implementation.
Time for each presentation should be increased to elicit extensively detailed information from groups.
The options were quite confusing during the [Delphi] poll. Took a long time during the workshop to re-define the options.

## 8. Conclusions 

This Project Report aimed to describe how an Integrated Assessment approach was used to study ASGM activities in Ghana. In doing so, the activities and approaches described and implemented here helped increase understanding of problems associated with the sector, particularly within and across disciplinary lines. In addition, the project enabled consensus building and included effective translation of scientific findings to stakeholders including those in decision-making positions. 
